# Colon Disease Diagnosis with Convolutional Neural Network and Grasshopper Optimization Algorithm

**DOI:** 10.3390/diagnostics13101728

**Published:** 2023-05-12

**Authors:** Amna Ali A. Mohamed, Aybaba Hançerlioğullari, Javad Rahebi, Mayukh K. Ray, Sudipta Roy

**Affiliations:** 1 Department of Material Science and Engineering, University of Kastamonu, Kastamonu 37150, Turkey; amnaalkmati@gmail.com; 2 Department of Physics, University of Kastamonu, Kastamonu 37150, Turkey; aybaba@kastamonu.edu.tr; 3 Department of Software Engineering, Istanbul Topkapi University, Istanbul 34087, Turkey; 4 Department of Physics, Amity Institute of Applied Sciences, Amity University, Kolkata 700135, India; mkray@kol.amity.edu; 5 Artificial Intelligence & Data Science, Jio Institute, Navi Mumbai 410206, India

**Keywords:** colon disease diagnose, convolutional neural network, grasshopper optimization algorithm, machine learning

## Abstract

This paper presents a robust colon cancer diagnosis method based on the feature selection method. The proposed method for colon disease diagnosis can be divided into three steps. In the first step, the images’ features were extracted based on the convolutional neural network. Squeezenet, Resnet-50, AlexNet, and GoogleNet were used for the convolutional neural network. The extracted features are huge, and the number of features cannot be appropriate for training the system. For this reason, the metaheuristic method is used in the second step to reduce the number of features. This research uses the grasshopper optimization algorithm to select the best features from the feature data. Finally, using machine learning methods, colon disease diagnosis was found to be accurate and successful. Two classification methods are applied for the evaluation of the proposed method. These methods include the decision tree and the support vector machine. The sensitivity, specificity, accuracy, and F1Score have been used to evaluate the proposed method. For Squeezenet based on the support vector machine, we obtained results of 99.34%, 99.41%, 99.12%, 98.91% and 98.94% for sensitivity, specificity, accuracy, precision, and F1Score, respectively. In the end, we compared the suggested recognition method’s performance to the performances of other methods, including 9-layer CNN, random forest, 7-layer CNN, and DropBlock. We demonstrated that our solution outperformed the others.

## 1. Introduction

Image processing methods are usually used for colon disease diagnosis, and careful learning is needed to analyze these images [1,2]. The image denoising during the image pre-processing steps can reduce the feature effectiveness, but the classifier methods can be mistaken, so the results will not be good. Additionally, some filters cannot give us the optimum results after image processing due to losing the features. In this paper, an attempt has been made to combine machine learning processes, such as combining learning of the neural network with the training of humans in order to reveal the areas of colon disease. The convolutional neural network has been used with AlexNet, SqueesNet, ResNet-50, and GoogLeNet in the proposed method’s first step to extract features from photos of colon illness. The feature numbers’ high values in these photographs make it inappropriate to utilize them in machine learning to categorize the patient’s condition; therefore, reducing the number of features is needed. Using the grasshopper optimization process, we reduced the number of characteristics. This process was selected because it is one of the best techniques for choosing features that quickly converge the fitness function. The support vector machine (SVM) and decision tree (DT) were employed in the classification step. The F1Score methodology and the confusion matrix were used to assess the outcomes. Globally, many people lose their lives as a result of many kinds of cancers. One of the most dangerous cancers is colon cancer. This type of cancer has been detected mostly in older people, especially women. For this issue, it is vital to find methods and ways to reduce cancer problems worldwide. This paper introduces an automatic process to find and classify colon cancers from histopathological images. 

Improving the classification accuracy is the primary goal of this study, which can be achieved by employing the best feature-selection strategy based on metaheuristic techniques. 

## 2. Literature Review

Random forest processes, support vector machines, CNNs, and Bayesian networks are the most widely used machine learning algorithms for cancer diagnosis. Mammogram pictures can be utilized to identify breast cancer using support vector machines and the random forest technique, as demonstrated by Bazazeh and Shubair [3]. In another study, Alam et al. [4] used support vector machines and computed tomography scans for lung cancer classification.

CNNs were recently used to help the cancer diagnosis process. Grayscale mammography pictures were used by Tan et al. [5] to detect breast cancer, for which they used CNNs. Moreover, Godkhindi and Gowda [6] created methods to detect colon cancer with the help of grayscale computed tomography colonography pictures using random forest processes, CNNs, and K-nearest neighbor (KNN) algorithms. A 3D CNN was utilized by Chiang et al. [7] to enhance the identification of breast cancer using 3-D ultrasound pictures.

Hyperspectral imaging involves new images as a tool for medical diagnostics as it becomes increasingly common in commercial and research applications. According to Lu and Fei, hyperspectral imaging is used for diagnosing diseases and as image-guided surgeries [8]. Moreover, they discussed several hyperspectral identification systems to test various malignancies, including breast, cervical, prostate, skin, and stomach cancers. Numerous cancer detection applications deal with the visible portions of the spectrum, specifically blue and green wavelengths. For example, it was found that the spectrum’s blue–violet region (390–450 nm) was strongly linked with colon cancer, according to research by Leavesley et al. [9].

Some new, non-invasive cancer detection methods have been developed when machine learning was combined with hyperspectral imaging [8,9,10]. For feature reduction with the help of PCA, the methods for hyperspectral data analysis were improved. For this purpose, minimal-spanning forests and support vector machines have also been applied. Linear discriminant analysis and support vector machines are used for hyperspectral colon cancer diagnosis, as Rathore et al. [11] mentioned. Lu et al. [12] used support vector machines with spectral and spatial data to increase the effectiveness of non-invasive neck and head cancer detection methods. Pike et al. [13] employed tumor-bearing mice to evaluate the hyperspectral classification of head and neck cancers. Gopi and Reshmi [14] created a technique that combines support vector machines with minimum-spanning forests to identify cancers in many animals.

Moreover, with the help of neural network models, frequent hyperspectral imaging has been used to improve cancer detection methods. For example, Leavesley et al. [15] used hyperspectral fluorescence and ANN to categorize colon cancer images. In [9] investigated ANN-utilizing hyperspectral images to classify cancer data. Ma et al. [16] identified head and neck cancers using CNN and pixel categorization. They created a 16 × 16 patch by taking each pixel’s image spectra and feeding it into a CNN.

## 3. Material and Methods

**Figure 1:**
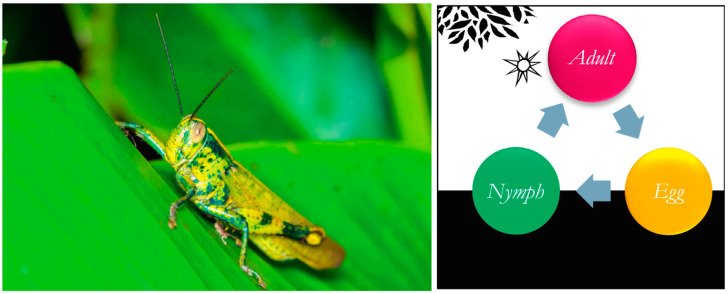
Real grasshopper and life cycle of a grasshopper [17].

Intelligent algorithms with natural inspiration are among the most effective methods for solving optimization issues. A smart population-based system called the grasshopper optimization algorithm mimics swarms of grasshopper social interaction and behavior. Saremi created this algorithm in 2017 [17]. Grasshoppers are among the pests that harm crops. Nymph and adult are the two stages that swarms of grasshopper s go through. A nymph in the first stage, without wings, feeds on plants in its path while slowly gaining freedom. The grasshoppers eventually develop wings to reach and generate swarms in the air for moving towards vast areas. Naturally, swarms of grasshopper s are separately observed because they produce enormous swarms of various kinds. The goal of the grasshopper was to find food sources through two different approaches, exploitation and exploration, each with a different illumination rate, according to Figure 1.

The movement of grasshoppers can be broken down into three categories: social interaction, gravitational force, and wind swing.

(1) Xi=Si+Gi+Ai

The value of the variable *Xi* in the mathematical equation indicates the ith grasshopper locus, whereas the variables *Si*, *Gi*, and *Ai* stand in for the social interactions among grasshoppers, the force of gravity acting on them, and the swing of the wind, respectively.

The equation Xi=r1Si+r2Gi+r3Ai clarifies that for providing a random approach, while *r*1, *r*2, and *r*3 are random numbers.

### 3.1. Distance and Movement

Here, $d_{ij}$ represents the distance between the *i*-th and *j*-th grasshoppers that can be calculated using the equation: $d_{ij}=\left|Xj-Xi\right|$. Here, *S* is used to define the largest social force using the following equation: $\hat{d_{ij}}=\frac{x_{j}-x_{i}}{d_{ij}}.$ It represents the carrier unit between the “*i*-th” and the “*j*-th” grasshopper, as given below:

(2) $$Si=\sum_{\begin{array}{c} i=1\\ i\neq j \end{array}}^{N}S\left(d_{ij}\right)\;\hat{d_{ij}}$$

Calculations can be conducted to determine the light absorption coefficient using the symbol *S*:

(3) $$Sr=fe^{\frac{-r}{l}}-e^{-r}$$

Here, *l* represents the light absorption factor, and f shows the source of the light intensity.

**Figure 2:**
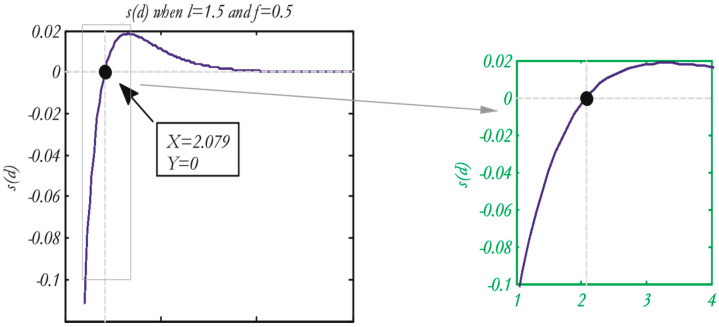
Function (*S*) (0 in the **right**) when *X* boundaries are 1−4 (**left**) for the function *S*, given that *f* = 0.5 and *l* = 1.5 [17].

Attraction and repulsion are two factors that might affect a grasshopper’s “social interaction”, as shown in Figure 2 of the function *S*, which displays the distances from 0 to 15.

**Figure 3:**
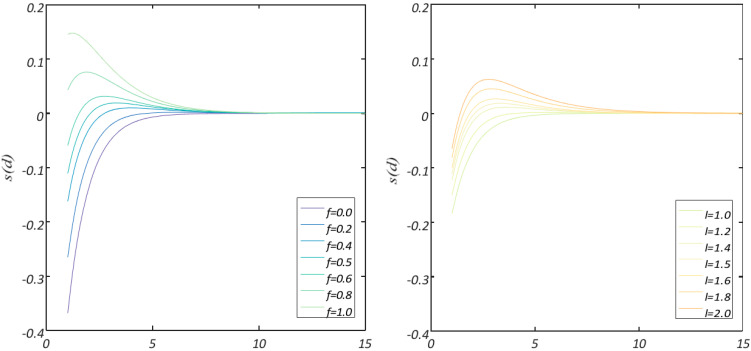
Function *S* in case of changing values of *f* or *l* [17].

The range of the repulsion process is 0-2.079. As demonstrated in Figure 2, no repulsion or attraction process was initiated in the comfort zone, where 2.079 is the distance between two grasshoppers. The unit of attraction first rises from 2.079 to almost 4, and then it “decreases gradually”. Figure 3 illustrates how altering the urging parameters (*l* and *f*) results in various social behaviors in artificial grasshoppers.

**Figure 4:**
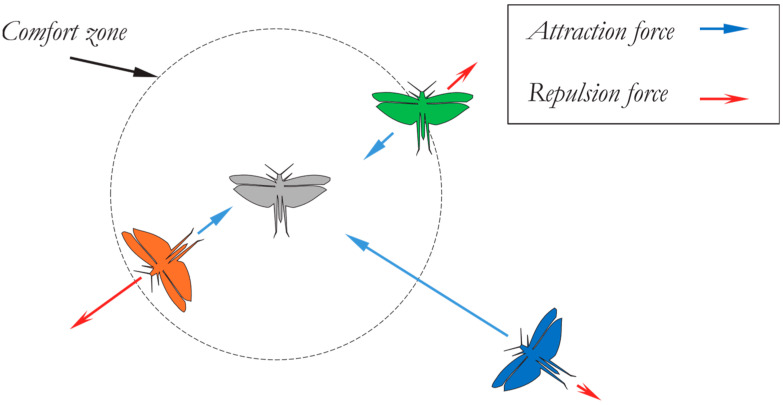
Primitive correction trends in a grasshopper swarm [17].

Occasionally, the values can be minimal in attraction and repulsion domains (either *l* or *f* = 1.0). It is important to note that *l* = 1.5 and *f* = 0.5 were selected from all values. Function S can divide the distances among the grasshoppers into different zones or stages, for example, the repulsion zone, the gravitational area, and the comfort zone. It illustrates interactions between grasshoppers, as shown in Figure 4, and also depicts the comfort zone. Moreover, with distances greater than 10, this function returns values constrained to 0, as shown in Figure 2 and Figure 3, which are on the same page. As a result, when there are great gaps between grasshoppers, this function is unable to calculate the appropriate forces to apply. 

In order to find the solution to this challenge, the distance between the grasshoppers within the range (1, 4) has been mapped. The function (*S*) for this period is depicted in Figure 3 (right) [17].

Equation (1), used for the calculation of variable *G*, is as follows:

(4) $$Gi=-g\hat{e_{g}}$$

At the Earth’s center, it represents g (gravitational constant) and $\hat{e_{g}}$ (unit vector). Through Equation (1), the variable A can be calculated as follows:

(5) $$Ai=u\hat{e_{w}}$$

Here, *u* represents steady erosion towards the wind $\hat{e_{w},}$ the unit vector.

Nymph grasshoppers lack wings; therefore, they are simply affected by the direction of the wind. The variables (*S*, *G*, and *A*) are replaced in Equation (1); thus, the equation will be as follows:

(6) $$Xi=\sum_{\begin{array}{c} j=1\\ j\neq i \end{array}}^{N}S\left(\left|X_{j}-X_{i}\right|\right)\frac{X_{j}-X_{i}}{d_{ij}}-g\hat{e_{g}}+u\hat{e_{w}}$$

*N* denotes the number of grasshoppers. 

The user model that rewards the swarm in free space is represented by Equation (6), and this equation replicates the interaction that occurs between the grasshoppers that make up the swarm.

(7) $$X\begin{array}{c} d\\ i \end{array}=c\left(\sum_{\begin{array}{c} j=1\\ j\neq i \end{array}}^{N}c\frac{ub_{d}-lb_{d}}{2}s\;\left(\left|X\begin{array}{c} d\\ j \end{array}-X\begin{array}{c} d\\ i \end{array}\right|\right)\frac{X_{j}-X_{i}}{d_{ij}}\right)+\hat{T_{d}}$$

In this case, *ub_d_* stands for the highest possible value in the *d*-th dimension, whereas lbd denotes the lowest possible value in the *d*-th dimension. $S\left(r\right)=fe^{\frac{-r}{l}}-e^{-r}\hat{T_{d}}$, where *Td* represents the value of dimension D in the target, which is considered to be the optimum solution, and factor c is lowering in order to decrease the dissonant area, attraction, and comfort zone.

The following equation shows that Factor (c) decreases from the comfort zone associated with the number of iterations:

(8) $$c=\mathrm{cmax}-l\frac{\mathrm{cmax}-\mathrm{cmin}}{L}$$

where l is the current frequency, cmax and cmin are the highest and lowest values, respectively, and L is the maximum cumulative number of repetitions. 

**Figure 5:**
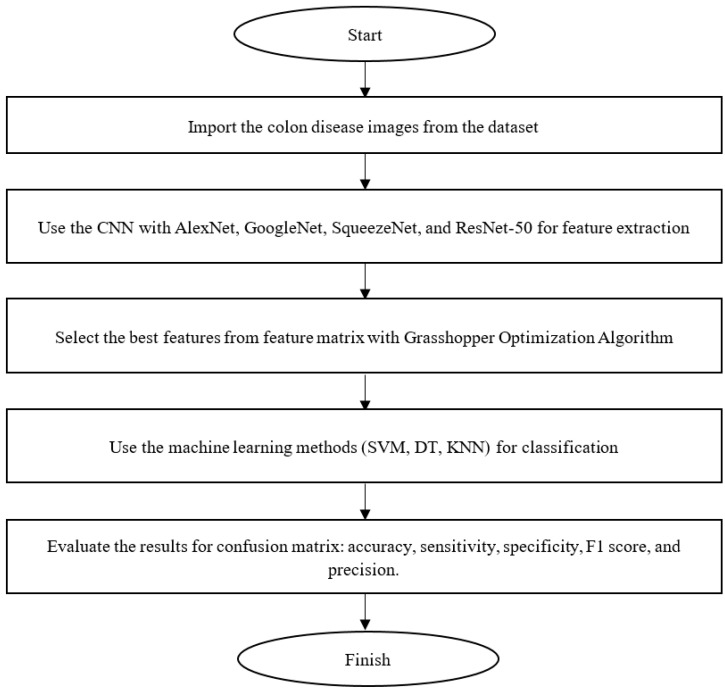
Flowchart of the proposed method for colon disease diagnosis.

Figure 5 shows the flowchart of the proposed method.

### 3.2. Dataset

This paper used the famous dataset “Lung and Colon Cancer Histopathological Images,” obtained from the open access dataset library. The dataset was obtained from:

https://www.kaggle.com/datasets/andrewmvd/lung-and-colon-cancer-histopathological-images (accessed on 12 April 2021).

The James A. Haley Veterans’ Hospital in Tampa, Florida, was the location where the data for this collection were gathered. The authors have largely gathered 1250 photos of cancer tissues from pathology glass slides, with a total of 250 photographs representing each form of cancer. They employed image augmentation techniques to rotate and flip the original photographs under different conditions, and as a result, they increased the size of the dataset to 25,000 images (with 5000 images in each class). Before applying the enhancement procedures, the photographs were cropped to a size of 768 by 768 pixels in order to make them square. The original dimensions of the images were 1024 pixels wide and 768 pixels high. All of the photos that are included in the collection have been validated, comply with the Health Insurance Portability and Accountability Act (HIPAA), and are available for no cost to users.

The data consist of 25,000 histopathology pictures in five classifications. Each image is a jpeg file, stored in the form of a 768 × 768-pixel image.

Overall, 750 images of lung tissue (250 lung adenocarcinomas, 250 lung squamous cell carcinomas, and 250 benign lung tissues) and 500 colon tissue images (250 colon adenocarcinomas and 250 benign colon tissues) were created using the valid samples of HIPAA-compliant sources and augmented to 25,000 using the Augmentor package.

In total, 25,000 color photos were found in five groups of 5000; each makes up the collection. A 1.85 GB zip file named LC25000.zip contains our dataset [18]. After unzipping, the two subfolders colon_image_sets and lung_image_sets are available in the main lung_colon_image_set folder. In the subfolder colon_image_sets, two further subfolders can be found, including colon_aca (5000 photos of colon adenocarcinomas) and colon_n (5000 images of benign colonic tissues). Three further subfolders are included in the lung_image_sets subfolder: lung_scc, lung_n, and lung_aca, each containing 5000 photographs of benign lung tissues. The lung_aca subfolder has 5000 images of lung adenocarcinomas.

The dataset consists of five classes, each with 5000 images classified as: (1) benign lung tissue; (2) lung squamous cell carcinoma; (3) lung adenocarcinoma; (4) benign colon tissue; and (5) colon adenocarcinoma.

This paper used the last two datasets, Colon benign tissue, and Colon adenocarcinoma. These datasets depend on the colon histopathological images. This research used 10,000 images, including 5000 cancerous and 5000 non-cancerous images. The dataset has been described in Table 1.

**Figure 6:**
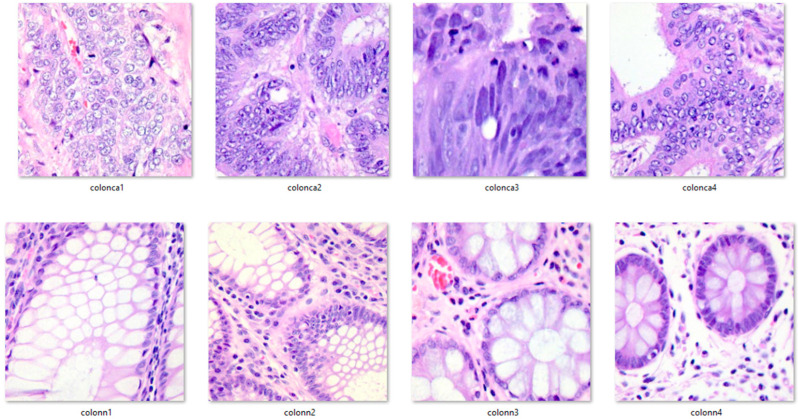
Sample images from the dataset.

Here are six histopathological image examples from the dataset (colon_n_ denotes a healthy image, and colon_ca_ denotes a colon cancer image). Figure 6 shows some of the images.

## 4. Results and Discussion

In this section, the results have been illustrated, and the proposed results have been compared with other methods and different types of CNN. Finally, the histopathological images have been used to implement the proposed method.

### 4.1. Data Set 

In this study, we used LC25000, and this dataset contains 5000 cancerous and 5000 non-cancerous images.

### 4.2. Evaluation

In this study, the classification of images is done using accuracy, sensitivity, precision, and *F*1 indices. Equations (9)–(12), respectively, define the criterion for each of the indicators [19,20,21,22,23]:

(9) $$Acc=\frac{TP+TN}{TP+TN+FP+FN}\times 100\%$$

(10) $$Recall=\frac{TP}{TP+FN}\times 100\%$$

(11) $$precision=\frac{TP}{TP+FP}\times 100\%$$

(12) $$F1=2\times \frac{Recall\times precision}{Recall+precision}\times 100\%$$

These indicators are calculated using True Negative (*TN)*, True Positive (*TP*), False Positive (*FP*), and False Positive Negative (*FN*). 

### 4.3. Analysis

Support vector machines and decision trees have been utilized in this study to categorize colon illnesses. The suggested approach has also been applied to several types of artificial neural networks. The number of iterations in the suggested method is 80, with a population size of 25 for the grasshopper optimization algorithm to choose the optimal characteristics. This study was simulated using MATLAB 2022a environment and an Intel Core I7 processor with a 4 GHz CPU.

#### 4.3.1. Objective Function Analysis

**Figure 7:**
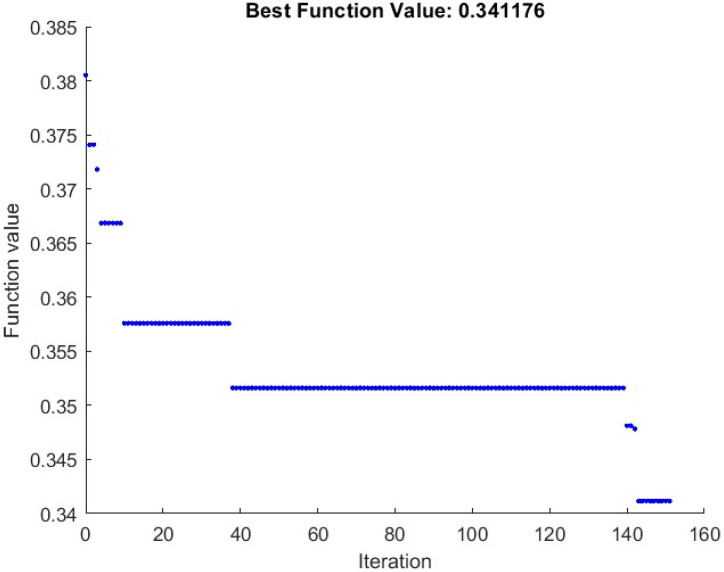
Reduction of feature selection function in terms of the GOA algorithm iterations.

One way to assess the proposed strategy is to calculate the value of the objective function of the feature selection. Figure 7 shows the average feature selection function (12) diagram for the photos. The feature selection objective function shows that it tends to become less repetitive compared to the GOA algorithm. This decrease shows that the GOA algorithm properly categorizes the images by choosing the best and the most appropriate feature vector for the neural network. According to analysis, two factors contribute to the objective function’s falling trend. The shrinkage of feature dimensions is the first justification. The second cause is the decrease in colon-disease-related image categorization errors.

Analysis reveals that the likelihood of discovering the optimal feature vector increases when the number of feature vectors increases because the goal function falls from 0.05481 to 0.00649. This reduction is around 8.44-fold.

#### 4.3.2. Classification Accuracy Analysis

**Figure 8:**
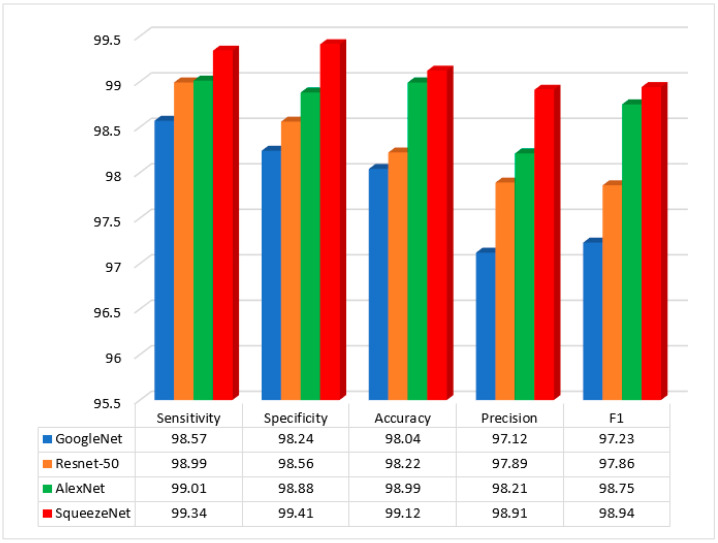
Results of the proposed method for different CNN methods.

Figure 8 illustrates the GOA algorithm’s accuracy in classifying photos of colon patients in an experiment. For implementation, four methods of CNN have been used and evaluated. These methods were GoogleNet, Squeezenet, Resnet-50, and AlexNet.

Analysis and evaluation reveal that the SqueezeNet approach performs quite well in terms of classification of image accuracy. The GOA algorithm’s careful selection of the feature vector led to an improvement in accuracy. Additionally, the Squeeze net approach reduces server-to-server transmission during distributed training. Selecting the best feature of GOA from SqueezeNet uses fewer errors in recognition of colon cancer and non-cancer data.

The GOA algorithm’s function is choosing the best feature vector for machine learning. The feature selection algorithm aims to choose the most significant characteristics from colon patient images for machine learning and training. In this experiment, GoogleNet’s accuracy is 98.12%, while SqueezeNet’s accuracy is 98.23%. When the feature vectors’ population grew, experiment 80 resulted in, on average, a 99.12% improvement in SqueezeNet’s colorectal dataset accuracy.

#### 4.3.3. Comparison 

Initially, the colorectal data set was selected to implement the proposed approach. Its effectiveness was assessed through images. According to Table 2, the suggested method’s sensitivity, specificity, and accuracy index findings compare with other metaheuristic methods such as ant colony optimization (ACO), particle swarm optimization (PSO), Genetic Algorithm (GA), and gray wolf optimization (GWO) algorithms with same CNN based on SqueezeNet methods. The proposed method’s mean values for sensitivity, specificity, accuracy, and F1 score compared with other approaches for the two classes are given below:

According to the findings of the study, the proposed technique has an average sensitivity of 99.34%, a specificity of 99.41%, an accuracy of 99.12%, and an F1 score index of 98.94%. The sensitivity, specificity, accuracy, and F1 Score of the suggested technique have been compared with those of various other metaheuristic methods, and the proposed method has been found to have a superior performance in the analysis and classification of images of colon patients.

**Figure 9:**
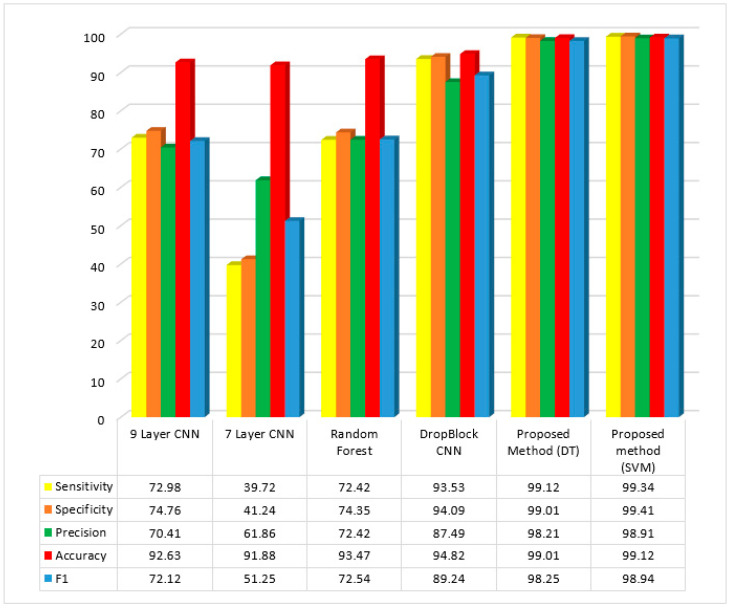
Mean index of sensitivity, specificity, precision, accuracy, and F1 in the proposed and other methods.

The GA technique has an accuracy index of 93.90%. This index, which is part of the suggested technique, currently stands at 99.12%. As seen from Table 2, the worst results are obtained from the GA algorithm. The obtained result for the GA algorithm were 97.18%, 95.79%, 93.90%, and 96.01% for sensitivity, specificity, accuracy, and F1Score, respectively. The outcomes were contrasted with those from several studies in this area, given in Table 3 and Figure 9:

Primarily, our proposed strategy has four indicators: accuracy, sensitivity, precision, and F1, for which some methods, such as DropBlock, 7-layer CNN, random forest, and 9-layer CNN, have been more successful in classifying colon cancer and non-cancer images. 

The proposed method’s sensitivity, specificity, precision, accuracy, and *F*1 in classifying images are 99.34%, 99.41%, 99.12%, 98.91%, and 98.94%, respectively. It was found that the 7-layer CNN method has the second-highest image classification accuracy, which is second to the proposed method; however, the 9-layer CNN method has the worst performance (91.88%). Moreover, the proposed method has also shown the highest sensitivity index value; however, the 7-layer CNN method has shown the worst performance. The proposed method has shown the best performance on the precision index, followed by the DropBlock CNN method with the highest sensitivity indexOverall, the proposed method has shown the best performance on the *F*1 index, whereas the 7-layer CNN method has shown the worst performance in this index.

## 5. Conclusions

The third leading cause of death by cancer worldwide is colorectal cancer (CRC). Adenomatous polyps (also known as adenomas), which are initially benign but may later develop into malignant polyps, may cause CRC. Routine screening to look for polyps is currently the recommended method for lowering CRC-related mortality, and colonoscopy is the screening method of choice. This paper attempts to combine machine learning, such as learning of the neural network, with learning and training in humans to reveal Cologne disease areas. In the proposed method, the GOA algorithm first increases the accuracy of the GOA technique for selecting the given feature by the optimization teaching and learning algorithm. Then, the disease-affected areas are separated by learning based on the neural network. In this paper, we have also used the component reduction method to improve the knowledge and information contained in the image. The proposed method has been compared with ACO, PSO, GA, and GWO methods. The best performance is obtained from the proposed method based on the SVM classification method.

## Figures and Tables

**Table 1:** Description of the dataset.

Image Type	Class ID	Class Title	Total Images
Colon Adenocarcinoma	0	Col_Ad	5000
Colon Benign Tissue	1	Col_Be	5000
Total images	10,000		

**Table 2:** Comparison of the Sensitivity, Specificity, Accuracy, and F1 Scores.

Method	Sensitivity	Specificity	Accuracy	F1 Score
ACO	97.21%	96.79%	95.58%	96.21%
PSO	97.89%	96.34%	94.67%	97.34%
GA	97.18%	95.79%	93.90%	96.01%
GWO	98.45%	97.18%	98.34%	97.99%
Proposed Method	99.34%	99.41%	99.12%	98.94%

**Table 3:** Comparison among different methods based on the average sensitivity, specificity, accuracy, precision, and F1 Score.

Reference	Method	Sensitivity	Specificity	Accuracy	Precision	F1 Score
[24]	Convolutional neural network and CLAHE framework	-	-	98.96%	-	-
[25]	Convolution neural networks	-	-	96.00%	-	-
[26]	Machine learning approach and deep-learning-based	96.37%	-	96.33%	96.39	96.38%
[27]	Deep Learning Method	97.00%		97.00%	97%	97.00%
[28]	Deep Learning with Bayesian–Gaussian-Inspired Convolutional Neural Architectural Search	93.00%	-	97.92%	97%	97.00%
[29]	Hybrid principal component analysis network and extreme learning machine	99.12%	99.38%	98.97%	98.87%	98.84%
[30]	Convolutional Neural Network	99.00%	-	99.00%	98.6%	98.8%
[31]	Transfer learning with class-selective image processing	-	-	98.40%		
[32]	Partial self-supervised learning	95.74%	80.95%	93.04%	95.74%	95.74%
Proposed Method	CNN based on SqueezeNet and GOA	99.34%	99.41%	99.12%	98.91%	98.94%

## Data Availability

The dataset was obtained from: https://www.kaggle.com/datasets/andrewmvd/lung-and-colon-cancer-histopathological-images.
